# Supporting families through paediatric brain tumour: Unmet needs and suggestions for change

**DOI:** 10.1002/pon.6136

**Published:** 2023-04-20

**Authors:** Kate Young, Christine Cashion, Timothy Hassall, Stuart Ekberg, Natalie Bradford

**Affiliations:** ^1^ Cancer and Palliative Care Outcomes Centre Centre for Healthcare Transformation Queensland University of Technology Brisbane Queensland Australia; ^2^ Centre for Children's Health Research Children's Health Queensland Hospital and Health Service South Brisbane Queensland Australia; ^3^ Children's Health Queensland Hospital and Health Service South Brisbane Queensland Australia; ^4^ School of Psychology & Counselling Queensland University of Technology Brisbane Queensland Australia

**Keywords:** brain neoplasms, cancer, oncology, paediatrics, psycho‐oncology, qualitative research

## Abstract

**Objective:**

From diagnosis and beyond, a paediatric brain tumour and its treatment impact the child and their family in a myriad of ways. While it is considered best practice to offer ongoing psychosocial support for all family members, there is little scholarly investigation of both families' experiences and the practical implications of offering such care. We aimed to explore families' experiences of paediatric brain tumour and their associated psychosocial health service needs.

**Methods:**

Families receiving care at the Queensland Children's Hospital in Brisbane, Australia, for a child (0–18 years) who had been diagnosed with a brain tumour between 2019 and 2022 were invited to be interviewed about their experiences. Using qualitative description, we analysed these interviews to identify families' unmet psychosocial health service needs and their suggestions for improvement.

**Results:**

Twenty‐three clinically and socially diverse families were represented. While parents/carers expressed gratitude for the care their child had received, most also described unmet needs for the broader family. We identified three primary needs to be addressed: (1) parents want accessible psychological/emotional support for themselves; (2) parents/carers want additional guidance to navigate the hospital setting to reduce uncertainty and loss of control; and (3) parents want support to minimise treatment‐associated trauma for their child.

**Conclusions:**

Our findings evidence the need for improved family‐centred psychosocial care within paediatric brain tumour care in Queensland, Australia. We propose a counselling and care coordination intervention to support parents/carers to care for themselves, their child, and their family through an extremely challenging experience.

## 1 INTRODUCTION

Brain tumour is the most common and deadliest solid tumour for children worldwide.^1^, ^2^ While 5‐year survival rates for most brain tumours in developed nations have improved in recent decades, around 26% of children diagnosed with a brain tumour will not survive.^3^ Families must then face living the rest of their lives without their child. When a child does survive, they and their families must navigate complex additional concerns because of the brain tumour and treatment at a pivotal point in physical and cognitive development.^4^, ^5^ These include seizures, cognitive deficits, and behavioural changes.

Few scholarly studies have considered the psychosocial experiences and associated health service needs of families who have a child diagnosed with a brain tumour. A recent qualitative review suggests each family member has varying unmet needs (see Young et al.^6^ and Young et al.^7^). Mothers, for example, report shouldering the burden of a lack of healthcare, policy, and social support in the often lifelong complex care required for their child, while adolescents and adult survivors emphasised a lack of care to address their mental health, particularly around body image.^6^ Quantitative studies suggest survivors of a brain tumour are more likely to experience depression and anxiety, and reduced self‐esteem and quality of life, later in life.^8^, ^9^, ^10^, ^11^ Parental distress—particularly for mothers—remains high years after their child's treatment is completed.^12^ Little is known about families' experiences soon after diagnosis and during treatment, with scant literature that explicitly explores and identifies what families want in terms of psychosocial health service delivery.^6^

In 2015, an international and interdisciplinary group of 80 stakeholders developed and published the Standards for Psychosocial Care for Children with Cancer and Their Families (see Weiner et al.^13^). These standards advocate for ongoing psychosocial support for all family members from the time of the child's diagnosis of cancer and beyond, recognising the complex interplay between the wellbeing of the child, caregivers, and family unit.^14^ However, such standards may be difficult to implement due to little use of standardised psychosocial assessment measures, and a lack of funding for associated services and integrated care for all family members.^15^, ^16^, ^17^ There is scant international research on the implementation of such standards or associated interventions, particularly with brain tumour where mortality is high and treatment involves many short and long‐term biopsychosocial impacts for the child and family.^18^ To the best of our knowledge, only one study with young adults has considered the Australian context where the aforementioned barriers are present,^19^ in addition to the recent added complexities of the Covid‐19 pandemic.^20^

We aimed to explore the psychosocial care experiences and needs of all members in a family where a child has been diagnosed with a brain tumour, and how these may be best addressed within the current care system^21^ in Queensland, Australia.

## 2 METHODS

This study is part of a larger project aimed at improving psychological, social, and economic outcomes for families who have a child diagnosed with a brain tumour. This research was initiated by the requests of families and clinicians at the Queensland Children's Hospital (QCH), and the study protocol was reviewed through the consumer advocacy group, Brainchild (brainchild.org.au). QCH is a tertiary public hospital located in the city of Brisbane in the state of Queensland, Australia. Despite the state's vast geographical dispersion, covering an area of 1.853 million square kilometres, all children diagnosed with a brain tumour in Queensland receive centralised oncology care through QCH, which is in the South‐East corner of the state. Our multidisciplinary research team includes clinical expertise in paediatric neuro‐oncology, and academic expertise in psychology, public health, and qualitative research methods.

The study protocol was approved by the hospital's Human Research Ethics Committee HREC/19/QCHQ/53816.

### 2.1 Participants and recruitment

Any family receiving care at QCH for a child who had been diagnosed with a brain tumour, or progression of a brain tumour, between 2019 and 2022 was eligible to participate. Children had to be under the age of 18 years at diagnosis of a malignant or non‐malignant brain tumour, or a sibling of a child who met these criteria. Parent/caregivers had to be at least 18 years old. Due to a lack of available translation services, all participants had to be English speaking.

Possible families were identified at the weekly Solid Tumour Multi‐disciplinary Team meetings by the clinical research nurse (Author B). If there was any concern regarding timing or appropriateness of approaching families, the neuro‐oncology clinical nurse consultant was consulted for advice. Recruitment to the study followed established principles, giving families time to make decisions about their involvement.^22^ If interested, Author B collected the participant's written informed consent and arranged a time of convenience to the family member/s to be interviewed.

### 2.2 Data collection

We had originally anticipated that Author A would conduct the interview as she is a trained qualitative interviewer and, not being a clinician, was likely a ‘neutral’ person for families to speak with about their care experiences (see Young et al.^23^ for original protocol). However, policies implemented during the initial phase of the Covid‐19 pandemic made it difficult for research staff to enter the hospital. As such, we pivoted to Author A training our research nurse, Author B, to conduct most interviews. A semi‐structured interview guide was developed, informed by our review,^6^, ^7^ the study aims, and participants' responses in prior surveys and/or interviews within the larger study. Participants were also encouraged to discuss anything relevant to their experience beyond this guide. Interviews could be conducted by telephone or online video call, in person, in hospital, or at the co‐located research facility. At the conclusion of each interview, participants were invited to email the study team if they wanted to arrange a later follow‐up interview. Data were collected from February 2021 to December 2022.

### 2.3 Data analysis

Interviews were transcribed verbatim through an external transcription service. We used qualitative description to identify families' unmet needs and suggestions for psychosocial care improvement at the system level. Qualitative description involves rich exploration of an experience or event where the researchers stay close to the data in both the analysis process and its presentation; it is particularly useful for medical research that seeks to describe patient and family experiences of a phenomenon.^24^, ^25^ Author A manually coded all transcripts using the software package, Nvivo, taking an inductive (data‐driven) approach. She then grouped these codes into themes and sub‐themes that were discussed and refined in several meetings with all authors who had varying familiarisation with the data having conducted none (Authors C and D), some (Authors A [*n* = 2] and E [*n* = 4]), or many (Author B; *n* = 21) of the interviews. Finally, Author A read through all transcripts in their entirety again to examine whether the final coding frame reflected the dataset. To aid translation through the language of intervention and implementation, we present the below key findings as thematic statements.^26^

## 3 RESULTS

Participant characteristics are presented in Table 1. Twenty‐three families are represented. Only one child elected to be interviewed and parents from four families participated in two interviews; for the purposes of this paper, only data from parents was included and data from multiple interviews with individual participants was pooled. Four children were in active treatment, two under observation with no treatment, one child had a terminal diagnosis, and the remainder were in remission with most having recently completed a treatment. We refer to participants as ‘parents’ unless the finding includes the one participant who is a relative and legal guardian of the diagnosed child. Each quoted participant is identified by their study identification number; brief descriptives for each are presented in Table 2.

**TABLE 1:** Interview and participant descriptives.

Variable	Outcome
Median interview length in minutes (range)	47 (20–107)
Interview mode	
In person	6
Telephone	10
Online video platform	11
Participant	
Mother	21
Father	2
Other guardian	1
Child sex	
Male	12
Female	11
Median months since diagnosis at time of interview (range)	20 (2–61)
Cancer grade	
Low	12
High	11
Treatments received	
Observation only	2
Surgery	19
Chemotherapy	9
Radiation	8
Immunotherapy	1
Location from hospital	
In Brisbane	16
Regional or rural	7
Household income	
<$70 000 per year	7
>$70 001 per year	11
Prefer not to say	2
Unavailable	3

*Note*: Socioeconomic data was collected from hospitals records where ethnicity is not routinely collected. Four participants referred to their family's ethnicity in their interview; these were Aboriginal Australian, Moroccan, Japanese, and South African heritage.

**TABLE 2:** Brief sociodemographic and clinical description for each quoted participant.

Study ID	Brief descriptive			
	Caregiver	Child's age at diagnosis	Child's sex	Tumour type
12	Mother	5 years	Male	Juvenile pilocytic astrocytoma
13	Mother	5 years	Male	Medulloblastoma
24	Mother	17 years	Male	Ganglioglioma
26	Mother	7 years	Female	Retinoblastoma
30	Mother	11 months	Female	Optic pathway glioma
38	Mother	5 years	Male	Medulloblastoma
43	Mother	3 years	Female	Juvenile pilocytic astrocytoma
69	Mother	4 years	Male	Dysembryoplastic neuroepithelial tumour
70	Mother	9 years	Female	Central nervous system neuroblastoma
83	Mother	3 years	Female	Retinoblastoma
86	Mother	4 years	Female	Juvenile pilocytic astrocytoma
91M	Mother	3 years	Female	Diffuse intrinsic pontine glioma
91F	Father	3 years	Female	Diffuse intrinsic pontine glioma
92	Mother	12 years	Male	Craniopharyngioma/pituitary tumour
113	Mother	4 years	Female	Retinoblastoma
115	Mother	14 years	Male	Pineal germinoma

Most parents/carers expressed gratitude for the care their child had received at the hospital. However, many also clearly described needs for themselves and their family that were not met by the current care system and gave suggestions for ways that this could be improved. Key points to action for intervention are outlined in Box 1.

**Box 1 Parents' and carers' suggestions to improve psychosocial family wellbeing in paediatric brain tumour.**

**Theme 1: Parents and carers want accessible psychological/emotional support for themselves.**

- Parent/carer mental health support integrated within child's care and routinely offered to all.
- Timing*—from diagnosis/during treatment/after treatment.
- Counsellor or psychologist* with a cancer background.
- Individual or group options.*

**Theme 2: Parents and carers want additional guidance about how the hospital works.**

- Provide a guide to families describing hospital layout, services, and processes, including:
  - Description of all hospital departments, staff within them, and how they relate to each other.
  - Describe and map out hospital facilities for example, cafeteria, toy library.
  - Describe day‐to‐day housekeeping within the hospital for example, timing of nursing shifts, food is available for parents/carers in this fridge, family toilet/shower location.
  - Map out nearby facilities and services for families who do not live near the hospital for example, the nearest grocery store.
  - List and describe all potential support services and financial assistance. Outline what services discontinue after time, departure from hospital, and/or treatment completing.
  - Foster parents' autonomy over their child's care for example, reminder that they can request a nurse comes back another time to allow their child to sleep.
- Paper document or mobile phone application.*
- Co‐develop with families to incorporate their experiential knowledge.

**Theme 3: Parents and carers want support to minimise treatment‐associated trauma for their child.**

- Provide parents with resources and support (e.g., occupational therapist, child co‐developed resources) to prepare their child for procedures, contextualising the need for them within the broader ‘story’ of their child's illness.
- Support child to take control of aspects of their care, as appropriate.
- Clinicians actively recognise and incorporate the expertise of parents, where possible.
- Be led by the child—not all want to know what is about to happen or why.

*Dependent on the individual/family's preference and situation.

### 3.1 Theme 1: Parents and carers want accessible psychological/emotional support for themselves

Parents consistently discussed the need for access to psychological and emotional support for themselves, to (a) assist themselves to navigate a challenging and life‐altering experience and to (b) enable them to better care for their child:

We’re not the one that the hospital is caring for, but because we are the carers of our daughter because she cannot do it for herself, she is too little…having that kind of focused support for us means that we can care for her better

(ID 91F)

No parent or carer reported being offered a psychology or counselling service from within the hospital. Most parents expressed a need for psychosocial services to be routinely available or made more accessible to all parents, even if they did not perceive a need for themselves: *…we're fine, but a part of me does think … that someone else in our situation, who may have been struggling would find it quite difficult because … we've never had a follow up, like how are you guys going?* (ID 83). Few parents described receiving emotional or psychological support from a social worker; more often social workers were described as providing practical support to navigate hospital and social services (see Theme 2).

Some participants discussed accessing—or attempting to access—psychology or counselling services in their community, sometimes at the suggestion of a hospital staff member. Getting a referral from their GP (to gain subsequent Medicare rebates), locating a suitable psychologist or counsellor, and attending sessions was described by parents as being laborious while managing the complexities of their child's treatment: *you need to talk to your GP to then start your mental health plan to then try and get to see a psychologist … just finding the time and the brain space …* (ID 91M,F). Some parents had access to psychology or counselling services through their paid employment though not all accessed this.

The preferred timing for service delivery varied for parents. Some wanted support to cope with the enormity of their child's diagnosis and treatment at the same time they were experiencing it: *the day* (our daughter was diagnosed) *our world just dropped … and from that day, I think, my husband and I really could've used some kind of counselling* (ID 70). One single mother wanted this to be in hospital ‘*so I don't have to move from* (my child)’ (ID 12), while another hadn't had any support because ‘[child] *is always with me … you can't say the things that you're thinking because* [child] *is sitting right there*’ (ID 115). Others stated they would best benefit from a service offered when their child was in remission, when they had the ‘*opportunity to process what had happened*’ (ID 24) and for those who ‘*at the time do fine and get through but afterwards … break down a bit*’ (ID 86). This was also when they now faced anxiety around tumour recurrence: *A headache is never just a headache … there's always that feeling in the back of your mind … what if it's back?* (ID 38).

Some parents stated a preference for a counsellor over a psychologist as the latter was perceived to ‘*medicalise things*’ when they ‘*just need help dealing with this*’ (ID 91M). A counsellor was often also more accessible by telephone or in their community, and sometimes part of a free community or charity service. A background in cancer was also described favourably by some parents as they ‘*don't have the energy*’ (ID 91F) to repeatedly retell their child's medical story: *She* [psychiatrist] *didn't understand cancer, so she was trying to ask me forty questions, what kind of cancer? It's like, I don't want to talk about that today* (ID 70). Opportunities for the sharing of people's stories—‘*connection for a shared experience*’ (ID 83)—with other parents in a similar situation were also wanted by some parents, and largely absent due to policies implemented during the Covid‐19 pandemic. Some parents of children with non‐malignant brain tumours pointed out that available supports didn't feel appropriate for them: *there are a lot of* *support* *groups for kids going through cancers, but … what you're going through, you feel like is a little bit different* (ID 69).

### 3.2 Theme 2: Parents and carers want additional guidance about how the hospital works

Many parents/carers discussed how navigating the hospital setting with little support compounded their distress, and increased feelings of uncertainty and loss of control: ‘*… it is kind of like starting a new job, like the number of things you need to know about suddenly is overwhelming*’ (ID 91M). Some parents, however, worked in hospitals (*I didn't have that same anxiety as my husband because hospitals are my workplace*—ID 24) or were ‘*already quite into the hospital life*’ (ID 30) due to having a child with a long‐term health condition/s.

Parents/carer described gaining more understanding of how the hospital worked through asking questions of staff, walking around the hospital, and having other parents tell them helpful information:

I just went to every level till I could figure out this hospital … It was overwhelming and, I'm like, I don’t know what I'm doing.

(ID 70)

We only found out about … the toy room where you can get loans and everything, coincidentally because a parent in the bed next to us told us on [child]’s third chemo cycle … we didn’t even know any of that existed.

(ID 38)

Families were typically cared for across several different hospital departments and moved through different wards. As one mother (ID 43) surmised, ‘*each* [ward] *was a little bit different in how they ran*’ and ‘*a one‐pager* [one page of written information] *for each*’ would be helpful for families, in addition to description of the ‘*the general flow of the hospital day‐to‐day,*’ including that ‘*as a parent you have the right to*’ request changes in procedures:

It wasn’t until we had been on the neurosurgical ward for a couple of weeks and got familiar with a few of the nurses [and they said] you can tell us if you don’t want us to come in for certain times.

Parents who lived far from the hospital described the additional burden of navigating travel and accommodation, and associated reimbursement through government subsidy schemes, for their child to receive treatment:

Through this whole process our biggest challenge has been dealing with patient travel … I can think of at least two occasions … when [husband and child] was discharged from the hospital … with no return flights arranged

(ID 113)

Parents/carers most often referred to a social worker as the staff member they thought would help them to access support from within and outside of the hospital; however, few found this to be their actual experience. One mother (ID 83), for example, shared ‘(social worker) *gave us our Red Kite bag* [a bag of practical items and information about childhood cancer from the charity, Red Kite] *… but outside of that* [we weren't informed of other services] … *the only time I ever spoke to the social worker … was when I asked a question*.’ Parents/carer described missing out on vital information about, for example, the existence and location of the welfare department, and resources to improve children's experiences (e.g., toy library, books, special beads) that may be ‘*silly things*’ to some but are ‘*what keep* [the child's] *day going*’ (ID 70). Families who lived far from the hospital and who were not familiar with the area had additional information needs, such as knowing the location of nearby grocery stores; it was difficult to source this themselves amid trauma when ‘*I don't have the mental capacity to … search Google maps*’ (ID 43).

An information binder (‘*like when you go to an Airbnb, except it's the worst Airbnb you can imagine*’—ID 43), mobile phone application, or ‘*induction package*’ (ID 91M) were described as possible modes to convey information about navigating the hospital to families. Suggested content, based on interviewees' reported experiences and explicit suggestions, is outlined in Box 1. Parents stated that while the request for such a guide may seem ‘*menial*’ (ID 43) to some, it ‘*would have made it a lot easier along the way*’ (ID70).

### 3.3 Theme 3: Parents and carers want support to minimise treatment‐associated trauma for their child

Several parents, mostly of younger children, discussed the need for improved support to minimise treatment‐associated trauma for their child. Specifically, parents reported their child was often hurried through procedures with little demonstrated consideration of the emotional impact on the child, and that parents felt unheard or dismissed when they suggested ways in which their child could be better supported. For example, one mother (ID 13)—for whom English is a second language—spoke about being repeatedly ignored when she requested that nurses not show or name to her son the general anaesthetic medicine as it greatly distressed him: *I cannot understand sometimes they* [nurses] *made me to feeling they don't care because if I say something, please can you don't say this, don't show this to* [child] *and you do this, it's very cruel.*

Parents often situated these experiences as a product of a system that was fragmented, under‐resourced (in part, due to the Covid‐19 pandemic), and that was not child or family‐centred:

He is four years old, he has cancer, nothing is in his control right now … the least you can do is give him five minutes … they would just say, but we don’t have time, we’ve got the next kid waiting

(ID 38)

Conversely, a few parents/carer only had positive things to say about how their child was treated: …*they included her immediately … she felt involved in the whole process … they really treat the kids individually and according to each of their needs* (ID 26).

Parents/carer outlined aspects of care that assisted in minimising their child's treatment‐associated trauma, as presented in Box 1. Of note, some parents of primary school aged children and younger discussed the helpful experience of receiving care from an occupational therapist (OT) within the hospital to manage their child's procedural anxiety: *she wasn't taking* [medication] *well initially … so we got into contact with OT and they gave us … some ideas on how to help her get used to it* (ID 30). This was also described by parents as being useful for the child and siblings to understand the broader context or ‘story’ of their illness:

We had a really good OT that worked with him and … [sister] … so she came up with [child]’s story, which explained what had happened … and the radiation and chemo he was going to have in a way that [sister] could understand so that she wasn’t scared and she actually took that story into school and took it to [child]’s class and spoke to his class about it.

(ID 38)

Those with adolescent children also spoke about giving their child control and autonomy over aspects of their treatment and the importance of having clinicians support this. Addressing the child's treatment goals and concerns was an essential element of this. One mother (ID 92), for example, spoke of her teenage son's concern about whether he would be able to play soccer after his surgery:

He was … told by one of the doctors that you will have an injury from this, but not given any information about what that might look like … [child] is soccer obsessed … and he would have lots of questions about, well can I play?

And another mother (ID 24) spoke of supporting her teenage son's decision to operate on his brain tumour despite her and her husband's fears:

We don’t want [you] chopped open, so no. I mean it was obviously what we were thinking, but not what we could possibly voice to him. [Helping] him understand all the positives and negatives and supporting him. Because this was his life.

## 4 DISCUSSION

This study explored the psychosocial care experiences and needs of families who have a child diagnosed with a brain tumour in Queensland, Australia. Our findings indicate the need for (1) improved access to psychological/emotional support for parents/carers, (2) increased guidance to navigate the hospital environment, and (3) improved support and care to minimise medical trauma for children.

### 4.1 Study limitations

Hospital restrictions related to the Covid‐19 pandemic, coupled with the nature of brain tumour and its treatment, inhibited our plans to include the experiences of children from their own perspectives.^23^ Recruitment was limited to a single Australian state, and those families who clinical staff deemed appropriate to contact and who could read and speak English. Nevertheless, parents of diverse families—in terms of both tumour type and treatment trajectory, and sociodemographic background—shared vital insight to their psychosocial care experiences and gave valuable suggestions for improvement.

### 4.2 Clinical implications

The gold standard for psychosocial care in childhood cancer includes psychosocial support for all family members from diagnosis and beyond.^13^, ^27^ Our findings suggest this is not the experience of families in Queensland, with parents reporting minimal assessment of, and support for, their psychosocial functioning throughout their child's entire tumour trajectory. This is consistent with research conducted in America^15^, ^16^, ^17^ and has been suggested by researchers trialling associated interventions in several countries including Iran, Sweden, Iceland, and Malaysia,^18^ and in Canada^28^.^19^

In our study hospital, with ∼280 referrals for childhood cancer each year, social work is universally offered to all families admitted to the oncology ward for 12 weeks only. There is designated psychological support for children but none available to parents/carers or siblings. While change at the hospital care system level is essential, a hospital‐adjacent intervention may sooner address the psychosocial needs of families. A nurse‐led counselling and education intervention to support carers of children with a brain tumour may improve caregiver psychosocial wellbeing (Theme 1).^29^ Including standardised psychosocial assessment that is shared with the treating team, and a care navigation and coordination component,^30^ may also improve families' access to hospital and community support services and resources (Themes 1 and 2).^28^, ^30^ To address paediatric medical trauma (Theme 3), there are several associated resources for families and clinicians to access, as summarised in De Young et al.^31^; these too could be shared within the aforementioned counselling and education intervention.

### 4.3 Conclusions

We can't yet cure cancer, but we can reduce associated distress for families. Consistent with other Australian and international studies, our findings clearly evidence the need for improved family‐centred psychosocial care within paediatric brain tumour care in Queensland, Australia. While increased government funding and systems‐level change is desperately needed, a hospital‐adjacent intervention may sooner address families' needs. We propose the development, implementation and evaluation of a counselling and care coordination intervention to support parents/carers to care for themselves, their child, and their family through an extremely challenging experience.

## AUTHOR CONTRIBUTIONS

Natalie Bradford, Timothy Hassall, and Stuart Ekberg designed and implemented the original project. Kate Young conducted all analyses and prepared the manuscript with assistance from all authors. Christine Cashion conducted all participant recruitment and most of the data collection with Natalie Bradford and Kate Young also contributing. All authors approve the final version of the manuscript for publication.

## CONFLICT OF INTEREST STATEMENT

The authors have no conflicts of interest to declare.

## Data Availability

The data is unavailable due to (1) the sensitive nature of the qualitative data used and difficulty in anonymising it, and (2) the participants of this study did not give written consent for their data to be shared publicly.
